# Prediction of Skin Color Using Forensic DNA Phenotyping in Asian Populations: A Focus on Thailand

**DOI:** 10.3390/biom15040548

**Published:** 2025-04-09

**Authors:** Gabriel Perez Palomeque, Supakit Khacha-ananda, Tawachai Monum, Klintean Wunnapuk

**Affiliations:** 1 PhD Program in Medical Sciences, Faculty of Medicine, Chiang Mai University, Chiang Mai 50200, Thailand; gabriel_perez@cmu.ac.th; 2 Department of Forensic Medicine, Faculty of Medicine, Chiang Mai University, Chiang Mai 50200, Thailand; tawachai.m@cmu.ac.th (T.M.); klintean.w@cmu.ac.th (K.W.)

**Keywords:** forensic DNA phenotyping, skin color prediction, single nucleotide polymorphisms, Asian populations, forensic genetics

## Abstract

Forensic DNA phenotyping (FDP) has emerged as an essential tool in criminal investigations, enabling the prediction of physical traits based on genetic information. This review explores the genetic factors influencing skin pigmentation, particularly within Asian populations, with a focus on Thailand. Key genes such as *Oculocutaneous Albinism II (OCA2)*, *Dopachrome Tautomerase (DCT)*, *KIT Ligand (KITLG)*, and *Solute Carrier Family 24 Member 2 (SLC24A2)* are examined for their roles in melanin production and variations that lead to different skin tones. The *OCA2* gene is highlighted for its role in transporting ions that help stabilize melanosomes, while specific variants in the *DCT* gene, including single nucleotide polymorphisms (SNPs) rs2031526 and rs3782974, are discussed for their potential effects on pigmentation in Asian groups. The *KITLG* gene, crucial for developing melanocytes, includes the SNP rs642742, which is linked to lighter skin in East Asians. Additionally, recent findings on the *SLC24A2* gene are presented, emphasizing its connection to pigmentation through calcium regulation in melanin production. Finally, the review addresses the ethical considerations of using FDP in Thailand, where advances in genetic profiling raise concerns about privacy, consent, and discrimination. Establishing clear guidelines is vital to balancing the benefits of forensic DNA applications with the protection of individual rights.

## 1. Introduction

Forensic DNA phenotyping (FDP) involves using biological samples to predict an individual’s physical appearance. This technique primarily relies on single nucleotide polymorphisms (SNPs) to estimate external visible characteristics (EVCs). It is a promising alternative in scenarios where short tandem repeats (STRs) are unfeasible due to sample degradation [1].

Moreover, FDP is increasingly considered reliable due to the documented unreliability of eyewitness testimonies, which are often inconsistent or inaccurate [2,3]. Common traits predicted through this method include eye color, hair color, and skin color, alongside other features like hair shape and freckles [4].

Historically, the most extensively studied EVCs have been eye and hair color. Studies predicting these traits have benefited from relatively homogeneous European populations, where genetic variation is more stable. The reason these two traits have been extensively researched, while skin color was not studied on a global scale until more recent years, lies in the unique challenges that skin color variation presents. Prediction studies of eye and hair color have gained accuracy due to the genetic consistency found in European populations. In contrast, skin color exhibits greater variation across different continental groups, necessitating studies in genetically heterogeneous populations worldwide. This highlights the importance of investigating Asian populations to enhance the accuracy of global skin color prediction.

The investigation of SNPs associated with skin color began in the late 1990s and early 2000s [5,6,7]. Although interest in using SNPs to predict physical traits emerged in the early 2000s, it did not gain significant traction until the end of that decade [8]. This delay in applying SNPs for the prediction of physical characteristics was primarily due to the fact that much of the genetic variation associated with these traits remained unidentified during the early stages of research [9]. It was not until the late 2010s that these predictive methods became more firmly established, benefiting from advancements in genetic research, large-scale sequencing technologies, and the expansion of comprehensive genetic databases. These innovations enhanced the accuracy and reliability of predicting skin color from DNA, marking a transition from preliminary research to more widespread and accepted applications in forensic science and related fields.

FDP has attracted significant interest within the forensic community as it could provide crucial investigative leads that contribute to identifying unknown individuals. While research in this field has made remarkable progress in European and African populations, there is a relative lack of studies examining the accuracy of appearance prediction in Asian populations, particularly those focused on Thailand. Given Thailand’s rich genetic diversity, studying SNPs related to skin pigmentation provides an exceptional opportunity to explore the accuracy and potential forensic applications of DNA phenotyping in this context.

Human skin color variation arises from a complex interplay between genetic factors and environmental influences, particularly UV exposure. This balance not only impacts vitamin D production and folate conservation but also demonstrates how these environmental forces have exerted selective pressure on the evolution of phenotypic traits over time [10]. In areas with low UV radiation, lighter skin pigmentation allows more UVB to penetrate the outer layers, promoting greater vitamin D synthesis [11]. Conversely, in regions with high UV exposure, darker pigmentation acts as a protective mechanism against DNA damage and folate degradation, illustrating an adaptive response to environmental pressures [12].

In light of these factors, melanin stands out as one of the primary contributors to skin color. Melanin is a natural pigment found in the skin, hair, and eyes, playing a crucial role in determining the range of colors observed in human populations. While melanin level is the primary determinant of skin pigmentation, its impact depends not only on its presence but also on the balance between its synthesis rate within melanocytes and its secretion rate from melanosomes, which together regulate the amount of melanin available for transfer to keratinocytes and its subsequent distribution within them [13]. Additionally, the size and shape of melanosomes contribute to skin pigmentation, with larger, more densely packed melanosomes generally associated with darker skin tones [14]. Melanin exists in two forms: eumelanin, which provides black and brown pigmentation, and pheomelanin, which contributes to red and yellow hues. The balance between these two types determines variations in skin pigmentation. Other factors, such as carotenoid intake [15] or hydration [16], have been found to be related to skin color.

In Asian populations, key SNPs, such as those in *oculocutaneous albinism type 2* (*OCA2)* [17,18], *dopachrome tautomerase* (*DCT)* [19,20], *KIT ligand (KITLG)* [21,22], and, more recently, *solute carrier family 24 member 2 (SLC24A2)* [23], have been identified as contributing to variations in skin pigmentation.

To provide a clearer understanding of the genetic variants discussed, Table 1 summarizes key variants in pigmentation-related genes and their associated alleles in Asian populations.

Although genome-wide association studies (GWAS) have been conducted in South Asian populations as early as 2007 [24], predictive studies on phenotypic traits in the Thai population remain largely unexplored, and the accuracy of such predictions has not been thoroughly investigated compared to European and African populations.

This review aims to evaluate the current state of knowledge regarding the prediction of skin color and the relationship between SNPs and skin pigmentation in Asian populations, with a specific focus on Thailand. By analyzing existing research and identifying gaps, it seeks to enhance the understanding of genetic factors influencing pigmentation and their implications for skin color prediction in this unique demographic.

**Table 1:** Variants in key genes associated with skin pigmentation in Asian populations.

Gene	Role	SNP	Alleles	References
*OCA2*	Transporter	rs74653330	G/A	[25,26]
		rs1800414	G/A	[17,18,25,26]
*DCT*	Enzyme	rs2031526	G/A	[20]
		rs3782974	A/T	[27]
		rs1407995	C/T	[17]
*KITLG*	Signaling molecule	rs642742	C/T	[21,22]
		rs1881227	C/T	[28]
		rs4073022	G/A	[22]
		rs428316	A/T	[22]
		rs11104947	G/A	[29]
*SLC24A2*	Transporter	rs10122939	G/A	[23]

## 2. Materials and Methods

A comprehensive literature search was conducted using a variety of keywords such as ‘Skin Color Prediction’, ‘Forensic DNA Phenotyping’, ‘Skin Color SNPs’, ‘Pigmentation in Thailand’, ‘Skin Pigmentation Genetics’, and ‘Skin Prediction’ across internationally recognized scientific databases, including PubMed, Scopus, and Web of Science. The studies were included in this review if they met the following criteria: (1) published in peer-reviewed journals from 2000 to 2024, (2) focused on the genetic basis of skin pigmentation in human populations, particularly in Asian populations with an emphasis on Thailand, while also considering other populations for comparative effects on pigmentation, (3) employed genome-wide association studies or candidate gene approaches with appropriate statistical validation, (4) included a sample size of at least 200 to ensure statistical power and reliability of findings, and (5) utilized robust statistical analyses to establish the significance of SNPs in skin pigmentation. However, the studies were excluded if they met any of the following criteria: (1) focused solely on animal models or non-human pigmentation genetics, (2) lacked sufficient statistical analysis to determine the forensic or biological relevance of reported genetic markers, and (3) were inaccessible in full text. The selection process involved an initial screening based on titles and abstracts, followed by a full-text review of studies that met the inclusion criteria. This approach enhances the reliability of the review by ensuring that only relevant and methodologically sound studies are considered.

## 3. Genetic Insights and Predictive Models for Skin Pigmentation

### 3.1. Genetic Background of Thailand

The genetic diversity of Thailand is believed to be significantly shaped by historical migrations, leading to the formation of distinct subpopulations. These migrations have contributed to the unique genetic landscape of the country, with each subpopulation carrying genetic signatures reflective of their origins and migratory patterns [30,31]. For instance, the Tai people, who migrated from southern China over a thousand years ago, form one of the largest ethnic groups in Thailand, significantly influencing the genetic makeup of the northern regions. Additionally, other ethnic groups, such as the Mon, Khmer, and Malay peoples, along with various hill tribes like the Karen and Hmong, have further contributed to the genetic diversity of the Thai population [32,33,34,35,36]. Within this genetic framework, predictive models for physical traits can be greatly enhanced by studying the genetic markers of these populations.

### 3.2. Advancements in DNA-Based Prediction of Pigmentation: Models and Techniques

The first DNA-based predictive model for eye pigmentation was developed by Walsh et al. in 2011, using a population of thousands of individuals from the Dutch demographic [37]. This model utilized SNPs from six genes previously identified as significant for iris pigmentation in a study by Liu et al. [38]. In 2013, the model was further refined with the introduction of the HIrisPlex system (available at https://hirisplex.erasmusmc.nl/; accessed on 15 January 2025) [39], which enabled the simultaneous prediction of both eye and hair color through the inclusion of 24 predictive DNA variants. Shortly after, in 2014, Maroñas et al. proposed a skin color predictive test, which employed 10 SNPs from eight genes known at the time to be associated with skin pigmentation [40]. Building upon these advances, the HIrisPlex system was expanded in 2018 to include 17 additional variants, thereby enabling the prediction of skin color alongside eye and hair pigmentation traits [41].

Although DNA-based prediction of pigmentation has demonstrated efficiency in forensic applications, its application remains limited, as previously published. Studies in other populations confirm that prediction accuracy decreases when models developed for one group are applied to genetically distinct populations, even within Europe [42]. SNP-based prediction has been studied in various populations, including Asian, African, and European groups. Notably, the study by Maroñas et al. demonstrated that certain SNPs related to skin pigmentation achieved high predictive accuracy, such as 87.6% for black skin and 95.7% for white skin. However, adding more SNPs further improved accuracy by 2–3% [40].

The HIrisPlex system, the first forensically validated tool for skin color prediction, has demonstrated its effectiveness on a global scale, achieving prediction accuracies of 74% for very pale skin, 72% for pale skin, 73% for intermediate skin, 87% for dark skin, and 97% for dark black skin [41].

Other studies utilizing the HIrisPlex system reported that the accuracy of predicting skin pigmentation in the European group ranged from 39% to 53% for dark skin, 67% to 70% for intermediate skin, and 92% for pale skin. However, they observed incorrect predictions for pale skin and noted that the HIrisPlex system had lower accuracy for light skin categories compared to dark skin categories. This finding highlights the need for identifying more predictive markers for light skin [43]. Also, the assessment of the IrisPlex-based multiplex for skin color prediction applied to a Portuguese population showed a positive predictive value and a negative predictive value of 93% and 96%, respectively, for all types of skin tone [44].

Seventy-seven SNPs from 37 genetic loci previously associated with human pigmentation were analyzed in 31 global populations, including Asian populations. The study found that the predictive value was 97% for light skin, 83% for dark skin, and 96% for dark-black skin. When classifying skin type according to the Fitzpatrick scale, the predictive values for very pale, pale, intermediate, dark, and dark-black skin were 74%, 72%, 73%, 87%, and 97%, respectively [45]. Moreover, researchers tested a skin color prediction model on 803 training samples. Among all samples, 600 (75%) yielded predictions with a low error rate of 1% (four errors). All four misclassifications involved dark-skinned individuals being incorrectly predicted as non-dark [46].

Even though there is limited information on SNPs related to physical traits in Asian populations, significant progress has been made in studying SNPs associated with ancestry. One notable tool is EurasiaPlex [47], which helps differentiate between European and South Asian ancestries using a specific set of SNPs. This tool combines 23 SNPs into a single test for improved genetic profile analysis and has demonstrated high accuracy in classifying individuals from these populations. In 2022, it was upgraded to EurasiaPlex-2, which specifically targets SNPs highly characteristic of South Asians, with minimal presence in other populations [48].

Similarly, another tool called JapanesePlex was developed to enhance the accuracy of ancestry classification specifically for the Japanese population [49]. This system utilizes a set of SNPs tailored to distinguish between Japanese and non-Japanese individuals, demonstrating the trend toward creating more refined genetic analyses for specific ancestral groups. Both EurasiaPlex and JapanesePlex exemplify the growing focus on population-specific SNP analyses in forensic and genetic research. Just as these tools have proven effective in forensic identification by leveraging unique alleles, similar approaches could be applied in Thailand to enhance the accuracy of forensic phenotyping. By focusing on the distinct genetic signatures of the various subpopulations in Thailand, future research can create more robust predictive models that account for local genetic diversity.

These predictive models are fundamentally based on genetic markers, primarily SNPs, which are identified using various molecular techniques, each with unique advantages and limitations.

PCR-based methods are commonly used for amplifying DNA regions containing SNPs, providing sufficient material for subsequent detection. While PCR alone is generally not suitable for direct SNP detection, studies have shown that modifications to conventional PCR protocols can enhance its utility, enabling the detection of specific SNP variants with greater sensitivity and specificity [50,51].

Notably, real-time PCR methods, such as those employing TaqMan probes, allow for SNP differentiation by exploiting fluorescence signals generated during the reaction, making it a valuable tool for targeted analyses [52].

SNP arrays are another widely used approach, relying on the hybridization of DNA to probes immobilized on a solid surface. This method allows for the simultaneous detection of multiple SNPs without prior PCR amplification, offering cost-effectiveness and scalability for large-scale studies [53].

Another commonly used technique to detect SNPs in samples is Single Base Extension (SBE), a variation of targeted sequencing [54]. While accurate and cost-effective, it is limited to detecting approximately 30 SNP markers per reaction [55]. In contrast, Next-Generation Sequencing (NGS) offers a more scalable and comprehensive approach, capable of detecting millions of SNPs across the genome in a single analysis. NGS fragments DNA, sequences these fragments in parallel, and reconstructs them computationally, making it ideal for degraded samples often encountered in forensic genetics due to its precision and sensitivity [56,57,58].

These methods are extensively applied in genome-wide association studies (GWAS) to uncover associations between genetic variants and phenotypic traits. By analyzing large genetic datasets, GWAS can pinpoint specific genetic loci associated with complex traits such as pigmentation, as well as other health-related characteristics, providing valuable insights into the genetic basis of these traits [59].

The earliest GWAS related to pigmentation was conducted by Sulem et al., involving nearly 3000 Icelandic individuals of European ancestry [60]. This study identified several significant SNPs associated with hair and eye pigmentation, as well skin sensitivity to sunlight and freckling. Among the notable findings were variants in two of the most extensively studied pigmentation genes, *melanocortin 1 receptor* (*MC1R*) and *OCA2*. Additionally, SNPs in other key pigmentation-related genes, such as *solute carrier family 24 member 4* (*SLC24A4*), *KITLG*, and *tyrosinase* (*TYR*), were also highlighted.

### 3.3. Genes Associated with Skin Pigmentation Variation in Asians

#### 3.3.1. *MC1R*

The *MC1R* gene encodes the seven-transmembrane G-protein-coupled melanocortin 1 receptor, which is located on the melanocytes. Upon binding to its ligand, alpha-melanocyte-stimulating hormone (alpha-MSH), this receptor increases intracellular cyclic adenosine monophosphate (cAMP) levels, subsequently activating protein kinase A (PKA) [61]. This signaling cascade ultimately promotes the production of eumelanin [62]. The *MC1R* gene is among the most extensively studied genes in relation to pigmentation. Variations within this gene have been shown to correlate with lighter skin and hair colors, particularly in European populations [5].

The primary hypothesis regarding this alteration in function suggests that polymorphisms within the coding region of the *MC1R* gene lead to the production of loss-of-function receptors contributing to lighter pigmentation compared to the wild-type receptors [63]. In Thailand, variations in *MC1R* have been identified only in specific northern Thai populations, specifically among groups such as the Shan, Lisu, and Black Lahu. In this demographic, nonsynonymous variants Val92Met (rs2228479) and Arg163Gln (rs885479) have been identified, but no significant changes in cAMP production were observed for these variants, suggesting that they may not play a crucial role in pigmentation variation [64].

**Figure 1:**
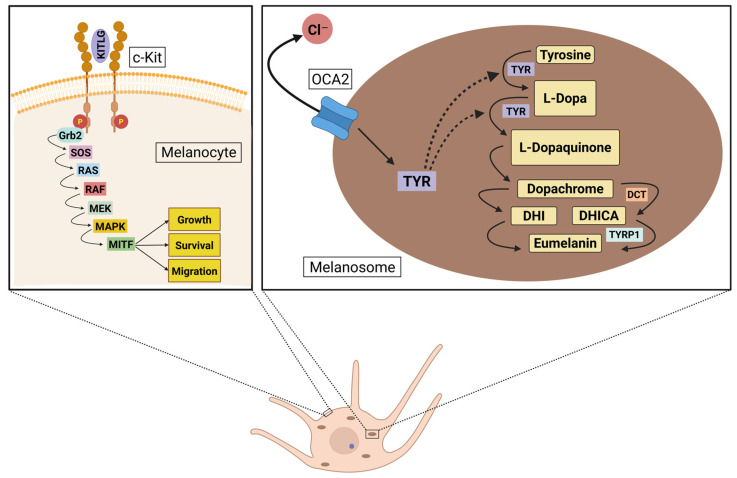
Schematic representation of melanocyte signaling pathways involved in pigmentation. The left panel illustrates the KITLG/c-Kit signaling cascade, which regulates melanocyte growth, survival, and migration through the MAPK pathway and activation of MITF. The right panel depicts melanin biosynthesis within the melanosome, highlighting the role of *OCA2* in chloride transport and *TYR* in catalyzing the conversion of tyrosine to melanin intermediates. Additional enzymatic steps mediated by *DCT* and *TYRP1* contribute to eumelanin production. Created in BioRender website. Perez, G. (2025) https://BioRender.com/s6erztw.

Since early research primarily concentrated on European populations, extensive information is available about variations in the *MC1R* gene within these groups. However, studies suggest that SNPs in this gene do not result in the same degree of pigmentary variation in other populations, such as those in Africa or Asia [65,66]. In contrast, other genes have been identified as key contributors to skin color variation in Asian populations. Among the key regulators of skin pigmentation, *OCA2*, *DCT*, and *KITLG* play indispensable roles in melanogenesis and skin development. *OCA2* is crucial for maintaining melanosomal pH and regulating tyrosinase processing, both essential for efficient melanin synthesis. *DCT* is a critical enzyme in the later stages of melanin production, directly influencing pigment type and stability. *KITLG* is fundamental for melanocyte survival, proliferation, and migration, ensuring proper melanocyte function and distribution in the skin. These genes have been consistently identified in genetic studies as core regulators of skin pigmentation and primary drivers of skin color variation across Asia [17,18,19,20,21,22,67]. The molecular mechanisms of melanogenesis, highlighting the involvement of these genes, are illustrated in Figure 1.

#### 3.3.2. *OCA2*

While variations in the *MC1R* gene have been shown to have limited relevance for pigmentation in Asian populations, several studies have established significant links between variations in the *OCA2* gene and pigmentation differences in various Asian groups. The *OCA2* gene encodes a transmembrane protein of approximately 110 kDa that spans the membrane 12 times and is predominantly localized in melanosomes [68]. This protein, referred to as the “P protein”, has been shown to play a crucial role in pigmentation. Research has shown that when the *OCA2* gene is expressed in heterologous systems, it is localized not only to melanosomes but also to lysosomes and late endosomes, suggesting a broader functional role than previously recognized [69].

The function of this P protein is believed to serve two main purposes. On one hand, it is responsible for the transport and processing of tyrosinase within the melanosome [70,71], the enzyme that catalyzes the hydroxylation of tyrosine to L-DOPA (L-3,4-dihydroxyphenylalanine) and the oxidation of L-DOPA to Dopaquinone, a key intermediate in melanin synthesis.

On the other hand, this same protein is known to participate in the ionic transport of crucial elements, particularly chloride ions (Cl^−^) [72,73], which are essential for maintaining melanosome stability and function. By regulating osmotic balance and pH, the P protein ensures optimal conditions for melanin synthesis and storage [74]. Tyrosinase, for instance, exhibits peak activity at a near-neutral pH, around 7, which is the environment found in mature melanosomes [75,76]. Deviations from this optimal pH range can lead to reduced enzyme efficiency or even denaturation. Additionally, disruptions in ionic transport can destabilize melanosomes and negatively impact the pigmentation process, contributing to disorders such as Oculocutaneous Albinism Type 2. In this condition, mutations in the *OCA2* gene lead to reduced melanin production [77,78]. In Caucasian populations, a more acidic environment within melanosomes has been suggested to suppress tyrosinase activity and inhibit melanogenesis [79].

A study conducted in Japan in 2022 [25] highlights the strong predictive power of specific SNPs in the *OCA2* gene for skin pigmentation traits in East Asian populations. The researchers identified rs74653330 and rs1800414 as key determinants of melanin levels, brightness, and yellowness. These SNPs, commonly found in East Asians, demonstrate significant associations with skin pigmentation, making them valuable for forensic applications. However, their predictive accuracy may vary in other populations due to differences in allele frequencies and genetic architecture, underscoring the need for population-specific validation.

Despite the lack of specific studies addressing the role of *OCA2* in skin pigmentation in the Thai population, one notable investigation was conducted by Yuasa et al. [26], which included 114 individuals from Bangkok as part of a broader sample. This research also examined these same alleles, OCA2*481Thr (rs74653330) and OCA2*615Arg (rs1800414), which are linked to hypopigmentation in East Asian populations. The study assessed the frequencies of these alleles in various groups, including Thais from Bangkok, alongside other East Asian populations like Evenkis, Oroqens, and Tibetans.

Even though studies on the role of *OCA2* in skin pigmentation in the Thai population are limited, some research has focused on SNP variants associated with eye color. For example, a study conducted in 2013 examined specific SNP variants, including rs7495174, rs4778241, and rs4778138, which were predominantly observed within this group [80].

#### 3.3.3. *DCT*

The *DCT* gene encodes the enzyme dopachrome tautomerase, which plays a vital role in melanin biosynthesis. *DCT* specifically catalyzes the isomerization of dopachrome into 5,6-dihydroxyindole-2-carboxylic acid (DHICA), a critical intermediate in the production of eumelanin [81]. This intermediate, in turn, functions alongside the enzyme TYRP1 (tyrosinase-related protein 1), which facilitates the oxidation of DHICA monomers, ultimately leading to the formation of eumelanin [82]. Together, *DCT* and *TYRP1* are essential in regulating melanin synthesis, influencing pigmentation in various tissues, including the skin and retina [83].

Back in 2007, researchers began to suspect that *DCT* might help explain some of the skin pigmentation differences observed in Asian populations. Notably, they discovered significant genetic variation in *DCT* between Chinese individuals and African and European populations, suggesting that local evolutionary factors in the Chinese population may have influenced pigmentation traits. Further investigation identified a specific SNP within the *DCT* gene, rs2031526, which exhibited signs of positive selection in the Chinese population [20]. The derived A allele was prevalent in Chinese individuals, while the ancestral G allele was found in European and African populations, pointing to recent evolutionary pressure. While it remains unclear if this SNP directly affects skin color, it underscores *DCT* as a potential contributor to pigmentation variation across populations.

Another study identified the rs3782974 variant in the *DCT* gene, which shows a significantly higher frequency of the derived A allele in Asian populations compared to the ancestral T allele [27]. While this SNP is located in an intronic region and does not result in an amino acid change, it may still influence gene regulation. However, no direct association between rs3782974 and skin pigmentation has been established.

In 2008, Alonso et al. identified several *DCT* gene variants that may contribute to pigmentation differences in the Asian population compared to African and European populations [19]. In addition to *DCT*, the study investigated variations in other key pigmentation genes, including *TYR* and *TYRP1*. Variations in these genes can significantly influence melanin synthesis and pigmentation traits, reinforcing the hypothesis that light skin in Europeans and East Asians evolved through different genetic pathways, illustrating the concept of convergent evolution. Years later, another study identified the intronic rs1407995 variant, where the derived C allele is more common in East Asian populations, while the ancestral T allele is more prevalent in European populations [17].

In Thailand, a recent investigation into a multifunctional octapeptide from Ganoderma lucidum revealed its role in melanogenesis through the upregulation of the *DCT* gene in melanoma cells [84].

#### 3.3.4. *KITLG*

The *KITLG* gene encodes the steel factor (also known as stem cell factor or KIT-ligand), which plays a critical role in the development and maintenance of melanocytes in adult skin [85]. This gene is also key in organ development by influencing the formation and structure of various tissues [86]. In the skin, *KITLG* is predominantly expressed locally in keratinocytes [87]. Additionally, it regulates cell proliferation and migration [88], processes that are vital for wound healing and cancer metastasis [89]. The receptor for KITLG, known as c-KIT, is a tyrosine kinase receptor that mediates these important signaling pathways [90].

The binding of the ligand to its receptor induces dimerization of KIT, activating its tyrosine kinase activity and leading to the autophosphorylation of tyrosine residues within the intracellular domain [91]. This activation promotes the recruitment of adaptor proteins, such as Grb2, which in turn initiate key signaling cascades, including the MAPK pathway, regulating cellular migration and survival [92].

Functional variations in *KITLG* are linked to pigmentation differences, while mutations in the gene are associated with skin disorders, including familial progressive hyper- and hypopigmentation (FPHH) and Dyschromatosis Universalis Hereditaria 2 (DUH2) [93,94].

One of the key *KITLG* variants associated with skin pigmentation, SNP rs642742, was identified in 2007 by Miller et al. [21]. This SNP, located upstream of the *KITLG* gene, exhibits significant variation between populations. The derived G allele of rs642742 is notably more prevalent in European and East Asian populations and has been linked to lighter skin tones. Individuals carrying this allele tend to have lower melanin levels, reflected in a lower average melanin index compared to those with the A ancestral allele, which is more common in populations of West African descent. This variant underscores the role of *KITLG* in the genetic mechanisms influencing skin color, particularly within East Asian populations, where the derived allele significantly impacts pigmentation characteristics.

In 2018, another significant study by Yang et al. [22] examined the evolutionary selection on *KITLG* and its effects on skin pigmentation, particularly among Eurasian populations. This research not only reaffirmed the association of the previously identified rs642742 but also reported additional variants, including rs4073022 and rs428316, linked to skin pigmentation in Han Chinese populations. The authors noted recurrent selective events in both the upstream and downstream regions of the *KITLG* gene, suggesting that adaptation to environmental factors such as UV radiation and winter temperature played a crucial role in shaping these genetic variations. Additionally, the variant rs1881227 has been associated with pigmentation variations across Asia and Europe [28].

Building on these findings, a significant GWAS conducted by Shido et al. [29] identified the intronic variant rs11104947, along with variants in *OCA2*, *BNC2*, and *DBNDD1*, as being associated with tanning ability in the Japanese population.

Despite these strong associations, *KITLG* variants have yet to be included in predictive models for skin color, pointing to a significant opportunity for future research. Exploring their role in forensic applications could lead to the creation of more comprehensive models that incorporate both genetic and environmental factors.

#### 3.3.5. *SLC24A2*

Another gene that has gained attention in recent years is *SLC24A2*, which has recently been linked to pigmentation in Chinese populations. This marks a notable shift, as it had not been previously associated with skin color.

This gene encodes a protein that functions as a calcium/cation exchanger, crucial for maintaining calcium balance within cells. Predominantly expressed in neurons [95], especially in the brain and retina, *SLC24A2* regulates calcium concentrations in response to light stimuli. Calcium regulation by *SLC24A2* is vital for processes such as neuronal signaling, synaptic plasticity, and potentially neuroprotection [96,97]. Interestingly, it also contributes to cellular functions like melanin production in melanocytes. Within melanocytes, fluctuations in calcium concentration in melanosomes can activate or inhibit tyrosinase activity [98,99]. Thus, through its diverse roles, *SLC24A2* is implicated in both neural activity and pigmentation pathways.

A study published in 2021 conducted a GWAS that examined skin pigmentation variation in Chinese populations [23]. The research found that the *SLC24A2* gene contains an intron variant (rs10122939) associated with skin color, where individuals carrying the G allele of this SNP have been linked to darker skin tones. While this allele is commonly found in the Chinese population, it is nearly absent in Europeans. The study proposes that variations in *SLC24A2* may influence pigmentation through several mechanisms. One hypothesis suggests that *SLC24A2* may moderate neuronal activity, which in turn influences melanocyte stem cells, potentially leading to changes in skin color. Alternatively, *SLC24A2* could alter the innervation of the skin, affecting characteristics such as skin thickness. As previously noted, early studies predominantly focused on European populations, causing this variant to be largely overlooked until more recent investigations.

This discovery is particularly relevant for the Thai population, as there are deep ancestral and migratory connections between Thais and East Asians, including the Chinese [28,100]. These interactions may have led to genetic overlap, particularly concerning pigmentation-related genes like *SLC24A2*. These findings align with observations from the studies discussed in this review, as illustrated by the SNPs listed in Table 2, which summarize the effects of various SNP alleles on skin pigmentation in different populations.

## 4. Forensic DNA Phenotyping in Thailand: Ethical Perspectives

New technologies bring new regulations, and FDP is no exception. As the field advances, important issues arise regarding privacy, consent, and the potential misuse of genetic information. The development of approaches like FDP requires the establishment of clear guidelines that protect individuals’ rights while allowing law enforcement to effectively use genetic data in criminal investigations. Even with forensic DNA profiling using STR markers, some studies have raised concerns about the possibility of genetic information revealing additional details about an individual that they have not previously consented to share [101,102].

In Thailand, DNA fingerprinting became widely adopted in criminal investigations during the early 2000s. While this led to significant advances in solving crimes, it also raised ethical concerns regarding the scope of its application and its potential impact on personal privacy [103]. The Central Institute of Forensic Science (CIFS), the main organization responsible for managing genetic databases for forensic identification purposes in Thailand, has played a key role in overseeing these advancements.

Efforts to establish a national DNA database by the CIFS began in 2004. Since then, significant progress has been made in collecting DNA samples from various sources, including crime scenes, prisoners, and crime suspects. As of 2019, it is estimated that the CIFS had accumulated over 180,000 samples, representing a considerable advancement in the collection of genetic data that could be utilized in forensic investigations and crime resolution [104]. However, despite this substantial collection of data, the national DNA database has not yet been formally established.

The use of genetic data, particularly SNPs, presents several ethical challenges and risks. Firstly, certain SNPs have been associated with predispositions to various health conditions, including cancer [105,106,107,108], as well as responses to certain medications [109]. The potential misuse of this information could lead to discrimination in various sectors, including employment and healthcare, where individuals might face unjust treatment based on their genetic predispositions. This practice raises significant ethical concerns regarding fairness and equity in access to necessary services. Secondly, this information not only affects the individual whose genome is being analyzed but also their relatives. The implications of revealing genetic information can extend to family members.

Another significant ethical issue arises from using genetic data to infer physical characteristics. Forensic DNA phenotyping could unintentionally reinforce social biases or contribute to racial profiling. In a country as ethnically diverse as Thailand, utilizing genetic information to predict skin color must be handled with extreme caution. Historically, darker-skinned individuals in Thailand have faced discrimination, and there is a legitimate concern that this prejudice could be aggravated by the inappropriate use of genetic data. Some studies have shown that East Asia, including Thailand, exhibits the highest levels of implicit bias against darker skin tones, particularly among women, and this bias often correlates with socio-economic and educational status [110]. Such implicit biases could spill over into forensic contexts, where genetic data could be exploited to predict skin color, potentially leading to profiling, discrimination, and the reinforcement of harmful stereotypes. Several experts have raised serious concerns about these risks, underscoring the urgent need for strict regulatory frameworks to prevent misuse [111,112].

Notably, as highlighted by earlier studies, skin color bias has been experimentally documented in various sectors, including criminal justice, where individuals with darker skin tones are often viewed with more suspicion [113]. In Thailand, where there is a growing market for skin-whitening products and a culture that often associates lighter skin with beauty and social status, the use of FDP could unwittingly contribute to this harmful cycle.

In addition, the use of FDP results to predict physical traits poses a substantial risk of influencing or even undermining eyewitness testimony. Although FDP predictions are inherently probabilistic, they may be perceived as definitive in judicial settings, leading to an undue emphasis on genetic evidence over other forms of testimony. Such overreliance could distort the interpretation of eyewitness accounts, potentially skewing how evidence is weighed in criminal trials. For instance, if FDP results suggest a particular skin color, this information may disproportionately overshadow contradictory eyewitness descriptions or other critical evidence.

Another critical issue is the excessive dependence on FDP, which could contribute to the so-called ‘CSI Effect’, where forensic evidence is perceived as infallible, thereby influencing judicial outcomes and increasing the risk of wrongful accusations [114]. A related concern is the ‘Kafka problem’, wherein even well-documented individuals can be described with varying physical traits, complicating forensic applications and raising concerns about subjectivity in phenotypic assessment [115]. While skin color prediction through FDP can be a valuable tool in forensic investigation, it also carries inherent risk. Without proper regulation, it could unintentionally lead to stigmatization, reinforce biases, or result in unfair treatment of individuals.

Therefore, strict guidelines and regulatory frameworks must be implemented to ensure that forensic DNA phenotyping is used responsibly. Importantly, forensic DNA phenotyping remains largely unregulated in many regions, with only a few European countries, such as the Netherlands, Germany, and Slovakia, having specific legal frameworks for its use [116]. In contrast, most other jurisdictions, including Thailand, lack clear guidelines, raising concerns about potential misuse. These safeguards should prevent FDP from being exploited or overinterpreted in legal contexts, ensuring that it remains a tool for investigation rather than a determinant of judicial outcomes. Additionally, the risk of discrimination based on physical appearance, particularly in sensitive areas such as skin pigmentation, should be addressed, ensuring the protection of vulnerable communities from racial profiling and genetic discrimination.

## 5. Limitations of Forensic DNA Phenotyping by Skin Color

FDP faces several challenges when applied to complex traits like skin color. Unlike STR profiling, which offers high polymorphism and enables individual identification, FDP provides probabilistic predictions that are prone to misclassification. For example, a study on eye color prediction in the Turkish population [117] demonstrated high accuracy for blue and brown eyes but a significant misclassification rate (78.94%) for intermediate eye colors. Given the polygenic and environmentally influenced nature of skin color, predictions are likely to face even greater challenges due to the combined effects of genetic factors and environmental influences such as UV exposure.

Furthermore, the widespread use of skin-whitening products in Thailand introduces an additional layer of complexity for FDP. As individuals have the ability to modify their physical appearance through various means, including cosmetics, medical treatments, and lifestyle choices, their observed phenotype may not always reflect their genetic background [118]. These products, driven by cultural preferences for lighter skin tones, may create a discrepancy between an individual’s genetically predicted skin pigmentation and their observed phenotype. This mismatch could potentially compromise the accuracy of FDP predictions in forensic investigations, particularly when attempting to reconcile genetic data with visible physical characteristics. When combined with the inherent difficulties of predicting traits across genetically diverse populations, these cultural practices underscore the need for cautious interpretation of FDP results. The societal pressure to alter natural skin tone thus presents both an ethical dilemma and a technical challenge for the application of phenotyping technologies in this region.

The polygenic nature of skin color, where multiple genetic variants contribute to pigmentation, increases the complexity of predictions and may lead to greater uncertainty [119]. While expanding the number of SNPs in predictive models may improve accuracy [46], it also introduces new challenges, as incorporating more markers amplifies the complexity of the prediction models.

Conversely, global skin color prediction models can achieve reliable results using a focused set of SNPs, even with degraded DNA often found in forensic contexts. However, considering multiple categorical probabilities rather than just the highest one improves the model’s ability to capture skin tone variation. Yet, further refinement and the identification of additional markers through large-scale studies are necessary to improve prediction accuracy [45].

Despite its challenges, FDP remains a valuable tool in forensic investigations, especially when genetic data are limited. Recent advancements, such as the use of likelihood ratios, provide statistical methods that better capture the complexity of non-genetic traits like pigmentation. By accounting for the relationships between variables, these models improve prediction accuracy, making FDP more reliable when used alongside traditional forensic methods [120].

## 6. Conclusions

The study of genetic variations in Asian populations holds significant potential for advancing forensic DNA phenotyping, particularly in the prediction of physical traits such as hair, eye, and skin color. Current predictive tools, such as HIrisPlex, have already demonstrated that SNPs related to skin pigmentation play a critical role in accurately predicting skin color. However, the database of this platform is primarily derived from European populations. This highlights the critical need for population-specific validation, particularly in regions like Thailand with complex genetic histories and admixture patterns. Expanding research to include Asian populations, where different genetic variants may play a more prominent role in pigmentation, is crucial for developing more inclusive and accurate prediction models.

Finally, exploring the genomics of skin color enhances our understanding of genetic variations in pigmentation and prompts us to confront the notion of race as a social construct rather than a biological reality. By recognizing that skin color differences arise from genetic diversity, we can apply FDP more ethically, developing models that better reflect the complexity of human traits.

## Figures and Tables

**Table 2:** Populations studied and the effects of SNP alleles on skin pigmentation.

Gene	SNP	Studied Population	Reported Effect	References
*OCA2*	rs74653330	Asians	A allele → Lighter skin	[25,26]
	rs1800414	Asians	A allele → Lighter skin	[17,18,25,26]
*KITLG*	rs642742	East/Southeast Asians	G allele → Lighter skin	[21,22]
	rs1881227	East Asians	T allele → Lighter skin	[28]
	rs428316	Chinese and Thai	T allele → Lighter skin	[22]
	rs11104947	Japanese	A allele → Tanning ability	[29]
*SLC24A2*	rs10122939	Chinese	G allele → Darker skin	[23]

## Data Availability

No new data were created or analyzed in this review.

## Abbreviations

The following abbreviations are used in this manuscript:

cAMP	Cyclic adenosine monophosphate
CIFS	Central institute of forensic science
DNA	Deoxyribonucleic acid
DUH2	Dyschromatosis universalis hereditaria 2
EVC	External visible characteristic
FDP	Forensic DNA phenotyping
FPHH	Familial progressive hyper- and hypopigmentation
GWAS	Genome-wide association study
MAPK	Mitogen-activated protein kinase
NGS	Next-generation sequencing
PCR	Polymerase chain reaction
PKA	Protein kinase A
SBE	Single base extension
SNP	Single nucleotide polymorphism
STR	Short tandem repeat
UV	Ultraviolet
